# Harnessing Nature: Computational and Experimental Insights Into *Ipomoea obscura* Metabolites for Bladder Cancer Therapy

**DOI:** 10.1002/cbdv.202500909

**Published:** 2025-09-02

**Authors:** Saravana Prabha Poochi, Rajamanikandan Sundarraj, Manikandan Vani Raju, Meenakshi Kaniyur Chandrasekaran, Rathi Muthaiyan Ahalliya, Chella Perumal Palanisamy, Gopalakrishnan Velliyur Kanniappan, Benod Kumar Kondapavuluri, Muthu Thiruvengadam

**Affiliations:** ^1^ Department of Biochemistry Karpagam Academy of Higher Education Coimbatore India; ^2^ Tianjin Key Laboratory For Modern Drug Delivery & High‐Efficiency, State Key Laboratory of Synthetic Biology and Frontiers Science Center for Synthetic Biology, School of Pharmaceutical Science and Technology, Faculty of Medicine Tianjin University Tianjin China; ^3^ Centre For Bioinformatics Karpagam Academy of Higher Education Coimbatore India; ^4^ Department of Biotechnology Karpagam Academy of Higher Education Coimbatore India; ^5^ Department of Dermatology Saveetha Medical College & Hospital, Saveetha Institute of Medical and Technical Sciences (SIMATS) Chennai India; ^6^ Department of Physiology Saveetha Medical College & Hospital, Saveetha Institute of Medical and Technical Sciences (SIMATS) Chennai India; ^7^ Department of Surgical Oncology Dr. D.Y. Patil Medical College, Hospital and Research Centre Pimpri Pune India; ^8^ Department of Crop Science, College of Sanghuh Life Science Konkuk University Seoul Republic of Korea

**Keywords:** bladder cancer, density functional theory, Ipomoea obscura (L.), MM/GBSA, molecular docking and dynamics, T24 cell lines

## Abstract

*Ipomoea obscura* (L.), a medicinal plant from the Convolvulaceae family, has been traditionally used to treat various ailments. This study evaluated the in vitro cytotoxicity of its ethanolic leaf extract against human bladder cancer T24 cells and investigated its phytochemical constituents through computational methods. The extract exhibited dose‐dependent cytotoxicity with an IC_50_ of 82.10 µg/mL, comparable to doxorubicin (75.04 µg/mL). Among 35 screened phytocompounds, kaempferol, oleic acid, and ursodeoxycholic acid showed strong binding affinity toward carbonic anhydrase IX (CA IX), with Glide docking scores ranging from −8.86 to −6.96 kcal/mol. These compounds formed stable Zn^2+^ coordination and hydrogen‐bonding interactions within the CA IX active site. Molecular dynamics simulations confirmed the stability of the ligand–protein complexes, and MM/GBSA analysis revealed favourable binding free energies. These findings indicate that the ethanolic leaf extract of *I. obscura* possesses anticancer activity against bladder cancer cells, potentially mediated through CA IX inhibition. Kaempferol, oleic acid, and ursodeoxycholic acid are likely contributors and warrant further investigation for their therapeutic potential in cancer treatment.

## Graphical abstract

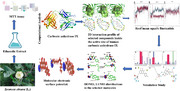

## 1 Introduction

Therapeutic plants employed in the field of medicine serve as cornerstones for valuable reservoirs of bioactive compounds. Recent studies have emphasized the role of plant‐derived metabolites in modulating key molecular targets in cancer therapy, making them vital candidates for drug development. For example, plant polyphenols and alkaloids have shown the ability to inhibit angiogenesis, induce apoptosis, and modulate oxidative stress pathways, which are crucial in cancer progression [1]. In addition, the structural diversity of plant secondary metabolites enables them to act as scaffolds for designing novel anticancer agents with improved specificity and reduced toxicity [2]. Phytomedicines derived from these plants are essential for the exploration and development of new pharmaceuticals for treating a broad range of ailments in both humans and animals [3]. Chemoprevention refers to the use of natural or synthetic chemicals to effectively inhibit, slow or reverse carcinogenesis. Recent findings demonstrate that chemopreventive agents can suppress tumor initiation and progression by modulating cellular redox states, regulating oncogenic signaling pathways (e.g., NF‐κB, PI3K/Akt), and inducing apoptosis and autophagy in malignant cells. For instance, plant‐derived polyphenols and saponins have shown promise in altering gene expression related to oxidative stress and inflammatory cascades, thereby offering therapeutic potential in bladder and other epithelial cancers [4, 5].

Chemopreventive agents generally have low toxicity and minimal side effects, functioning by neutralizing carcinogens and reducing their effects on cells [6, 7]. In addition to detoxifying carcinogens, these agents modulate antioxidant enzyme systems, regulate DNA repair mechanisms, and influence cell cycle arrest and apoptosis in transformed cells. Phytochemicals like flavonoids, alkaloids, and phenolics have been reported to exhibit chemopreventive properties by altering the expression of proapoptotic and antiapoptotic genes, restoring redox balance, and suppressing oncogenic transcription factors [8, 9]. Cancer incidence varies according to factors such as age, sex, geographical location, and socioeconomic status. As the population ages and risk factors become more common, the incidence of cancer has generally increased [10]. Bladder cancer is the tenth most common cancer worldwide, with approximately 550 000 new cases diagnosed each year [11]. Global cancer research shows that men are four times more likely to develop bladder cancer than women. However, female patients diagnosed with bladder cancer are at a higher risk of recurrence, progression, and cancer‐specific mortality than their male counterparts [12]. The conventional method for treating muscle‐invasive urinary bladder cancer involves a standard approach known as radical cystectomy, which includes a bilateral pelvic lymph node analysis [13].

Common plants and herbs have free radical‐neutralizing, anti‐inflammatory, and anticancer effects. As a result, several of these plants are often used to treat a variety of diseases. *Ipomoea obscura* (L.) is a tiny climbing perennial plant belonging to the Convolvulaceae family. It thrives in meadows, hedgerows, and wastelands in India. This plant is found in Africa, Asia, and several Pacific islands. It is a tiny climber with short, cordate leaves that grow as an undergrowth in hedges or low ground cover, up to 3000 ft above sea level [14]. The corolla was united with five petals and lovely flowers [15]. Ayurveda examined the medicinal properties of this fresh plant extract under various conditions, including open sores, pustules, and diarrhea. When mixed with gingelly oil (sesame oil), it is used to treat asthma and cold and dry cough. A paste made from the leaves is used for snake bites, hemorrhoids, ulcers, and inflammation. Moreover, the fruits and seeds act as cleansing agents, pain relievers, and remedies for respiratory issues, and also improve vision [16, 17, 18]. *I. obscura* is a small climbing perennial plant from the Convolvulaceae family, distributed across India, Africa, Asia, and the Pacific islands. Traditionally used in Ayurveda for conditions such as inflammation, ulcers, and respiratory ailments, recent studies have identified a range of phytochemicals in this plant, including flavonoids, alkaloids, and phenolic compounds with known pharmacological effects. Notably, compounds such as ipobscurines and calysteginines possess anti‐inflammatory, antioxidant, and anticancer activities. Prior research has demonstrated its anti‐tumor and COX‐2 inhibitory effects, suggesting a mechanistic link to inflammation‐associated cancers. These findings support the rationale for investigating *I. obscura* as a potential source of anticancer agents, specifically targeting human carbonic anhydrase IX (CA IX), a hypoxia‐associated enzyme overexpressed in bladder and other cancers. The phytochemicals present in this plant include flavonoids, steroids, phenolic acids, and essential oils. Notable pharmacologically active compounds found in the plant are the indole alkaloids ipobscurine A, B, C, and D, as well as the tropane alkaloids calysteginine B‐1, B‐2, B‐3, B‐4, and C‐1. Various compounds derived from the extracts of *I. obscura* exhibit anti‐inflammatory, antioxidant, anticancer, antimicrobial, nephroprotective, anti‐invasive, and antimetastatic properties [19, 20, 21].

Carbonic anhydrase is a member of the zinc metalloenzyme family, which comprises various isoforms that are associated with different levels of enzyme activity and kinetic properties [22, 23]. These isoforms, such as CA I, II, IX, and XII, display tissue‐specific expression and distinct structural characteristics, which influence their catalytic efficiency, substrate specificity, and physiological function. In particular, CA IX is membrane‐associated and predominantly expressed under hypoxic conditions in solid tumors, making it a strategic target for selective cancer therapy. Recent studies have highlighted the potential of sulfonamide and coumarin‐based inhibitors to selectively bind and inhibit CA IX while sparing off‐target isoforms like CA I and II, thereby minimizing systemic side effects [24]. Among the various isoforms, only two, CA IX and CA XII, have been identified as potential targets for cancer therapy [25]. The human CA IX enzyme serves as an endogenous marker for hypoxia and is overexpressed in several cancers, including bladder, head, lung, and renal cancer. Its overexpression is often correlated with poor prognosis, treatment resistance, and increased tumor invasiveness under hypoxic conditions. CA IX plays a critical role in maintaining pH homeostasis in the tumor microenvironment by catalyzing the reversible hydration of CO_2_, thereby enabling cancer cells to survive and proliferate in low‐oxygen conditions. Recent studies have demonstrated the efficacy of natural product derivatives and synthetic inhibitors that selectively block CA IX activity, resulting in apoptosis, reduced migration, and tumor regression in preclinical cancer models. In the context of bladder cancer, CA IX is markedly overexpressed in high‐grade tumors and is associated with poor differentiation, metastasis, and resistance to radiotherapy and chemotherapy. Hypoxic regions within bladder tumors promote the upregulation of CA IX through hypoxia‐induced factor‐1α (HIF‐1α) activation, facilitating extracellular acidification that supports tumor cell invasion and immune evasion. As a result, CA IX has emerged as a relevant biomarker and therapeutic target for bladder cancer, with several CA IX‐specific inhibitors currently under investigation for their potential to improve treatment response in this malignancy. It possesses chemomodulatory properties, making it a promising target for the development of novel anticancer drugs [26]. Under hypoxic conditions, the binding interaction between HIF‐1α and hypoxia‐responsive elements directly promotes upregulation of the CA IX enzyme [27]. These hypoxia‐responsive elements have a pro‐survival function by maintaining high intracellular and low extracellular pH, which helps prevent apoptosis and activates proteolytic processes, such as invasion, through various signal transduction pathways [28]. The overexpression of CA IX has been noted in various cancers and is associated with poor response to conventional chemotherapy and radiotherapy. Structural analysis revealed that CA IX consists of three domains: an N‐terminal domain, a C‐terminal domain, an extracellular catalytic domain, and a short intracellular tail. The N‐terminal domain is involved in protein‐mediated cell adhesion, whereas the C‐terminal domain plays a role in catalyzing the reversible hydration of CO_2_, producing bicarbonate ions essential for pH regulation [29]. Hence, in this study, we aimed to detect the possible bioactive constituents of *I. obscura* that may be responsible for its anticancer activity via CA IX inhibition through an in silico approach.

## 2 Results and Discussion

### 2.1 Anticancer Activity of Ethanolic Leaf Extract of *I. obscura*

**FIGURE 1:**
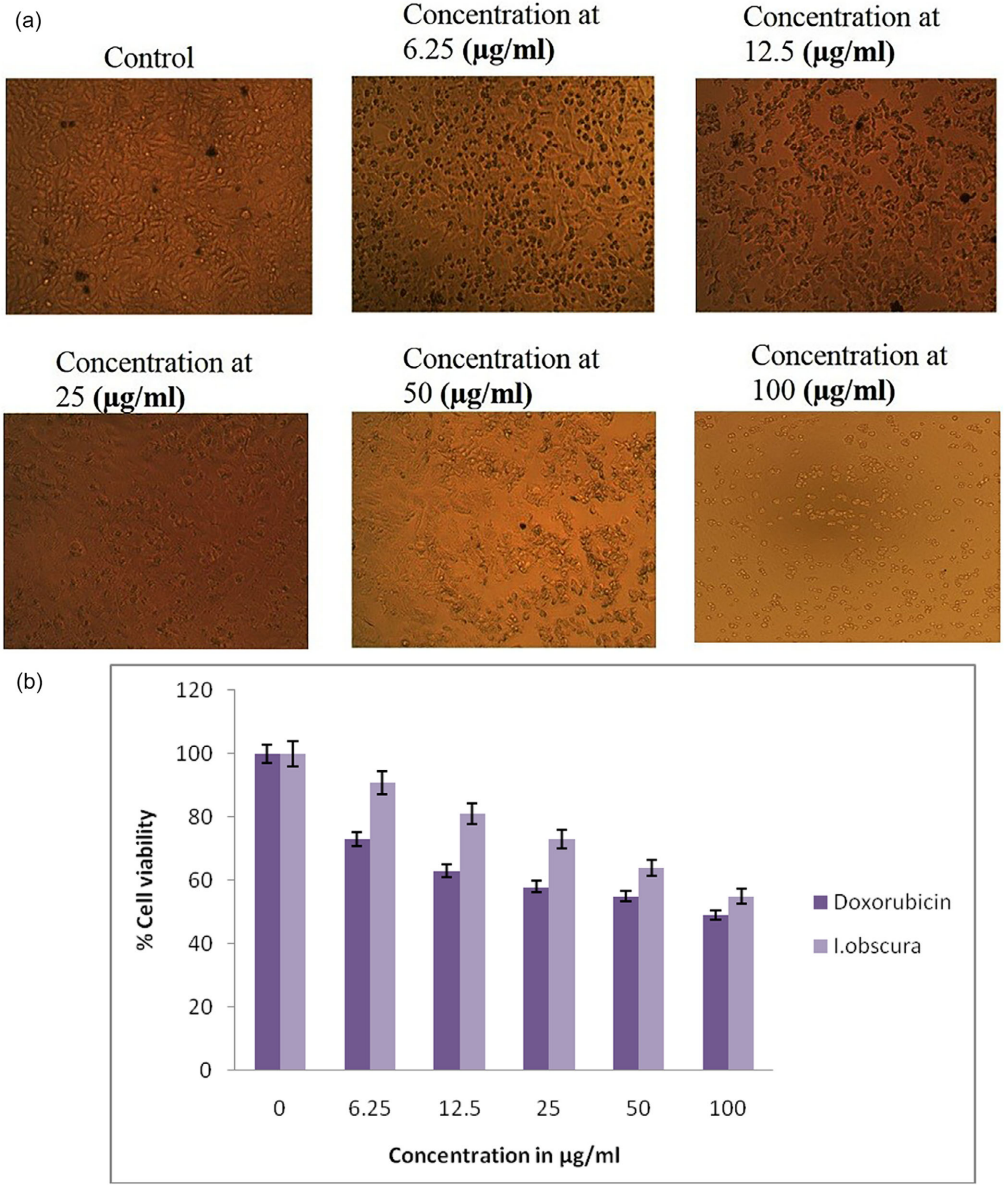
(a) Cytotoxicity analysis of T24 bladder cancer cell lines against different concentrations of *Ipomoea obscura*. (b) Effect of *I. obscura* on T24 bladder cancer cell line at different concentrations after 48‐h incubation.

Figure 1a shows a morphological analysis of the T24 bladder cancer cell line. The viability of cancer cells decreased with an increase in the extract concentration. Figure 1b shows the cytotoxic effect of the extract on the T24 bladder cancer cell line at different concentrations after incubation for 48 h. Doxorubicin hydrochloride was used as the standard drug. The susceptibility of cells to drug exposure was characterized by IC_50_ values. After the incubation of 48 h, T24 cells showed IC_50_ values of 75.04 and 82.10 µg/mL for doxorubicin hydrochloride and *I. obscura* extract, respectively. IC_50_ values at other time points were not calculated, although cell viability was monitored. Notably, normal (non‐cancerous) cell lines were not assessed in this preliminary study; thus, a selectivity index was not determined. This is recognized as a limitation and will be addressed in future work. The results indicated that the antiproliferative effect strengthens with an increase in drug concentration.

### 2.2 Molecular Docking

**FIGURE 2:**
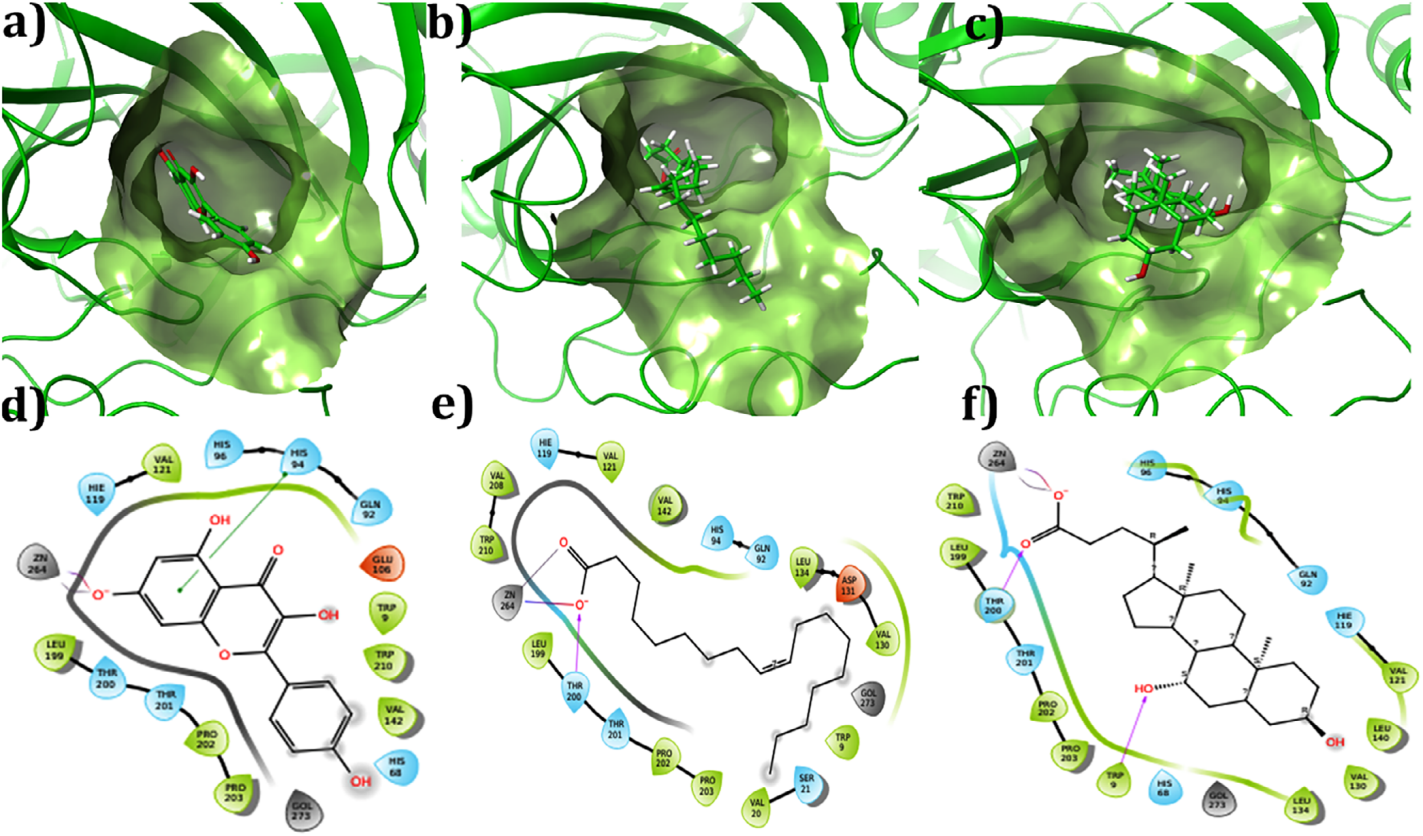
(a–e) Two‐dimensional interaction profile of selected compounds inside the active site of human carbonic anhydrase IX. (a, d) Kaempferol, (b, e) oleic acid, (c, f) ursodeoxycholic acid. In the two‐interaction diagram, the H‐bond, π–π stacking, metal coordination, and salt bridge interactions were denoted in pink, green, gray, and mixed colors dashed lines, respectively.

The binding conformation of the small molecules to the binding site of CA IX was predicted using molecular docking simulations. To validate the docking program, re‐docking was performed with the co‐crystal ligands in the experimentally predicted binding pocket of the protein. Based on the results, it was observed that the similarity between the docked orientation and experimental predicted orientation was about 0.16 Å, which confirms the docking protocol employed in this study. The docking results of the compounds with CA IX are shown in Table 1. The three compounds showed Glide score and Glide energy in the range of −8.86 to −6.96 kcal/mol and −33.66 to −31.04 kcal/mol, respectively. Kaempferol showed the highest Glide score (−8.86 kcal/mol) among the other compounds selected. Binding mode analysis revealed that the compound had one π–π stacking interaction with His94, with a bond distance of 4.80 Å. The carbonyl group of kaempferol showed metal coordination and salt bridge interaction with Zn264, with a bond distance of 2.05 Å, respectively. The Glide score of −6.94 kcal/mol and the Glide energy of −31.04 kcal/mol were noticed for the CA IX–oleic acid complex. The carbonyl group (C─O) of the compound established one H‐bond interaction with the amine group (NH) of Thr200, with a bond distance of 1.98 Å. In addition, the compound also established metal coordination and salt bridge interaction with Zn264 with a distance of 2.01 and 3.86 Å, respectively. The interaction pattern observed between ursodeoxycholic acid and CA IX showed two H‐bonds, Thr200 (C─O⋯HN; bond distance = 1.89 Å); Trp9 (HO⋯HN, bond distance = 2.27 Å) one metal‐coordination (C─O⋯Zn264, bond distance = 2.07 Å) and one salt bridge (C─O⋯Zn264, bond distance = 2.07 Å) interactions. The complex showed a Glide score of −6.96 kcal/mol and a Glide energy of −34.74 kcal/mol. Binding mode analysis of the compound inside the binding site of CA IX is shown in Figure 2. All molecules showed acceptable absorption, distribution, metabolism, and excretion (ADME) profiles (Table 2).

**TABLE 1:** Molecular docking results of *Ipomoea obscura* against human carbonic anhydrase IX.

S. no	Compound ID	Name of the compound	Glide score (kcal/mol)	Glide energy (kcal/mol)	Glide *E*_model_ (kcal/mol)
1	**5280863**	**Kaempferol**	−8.863	−33.661	−49.025
2	**445639**	**Oleic acid**	−6.994	−31.048	−35.271
3	**31401**	**Ursodeoxycholic acid**	−6.968	−34.747	−38.202
4	985	Palmitic acid	−6.903	−30.054	−31.373
5	867	Malonic acid	−6.416	−23.453	−30.095
6	54675777	Chlortetracycline	−5.807	−30.803	−33.382
7	8998	Tetritol	−5.439	−23.101	−23.687
8	54680690	Demeclocycline	−5.301	−31.029	−35.431
9	54675776	Tetracycline	−5.152	−30.764	−37.197
10	58	Butanoic acid	−5.073	−23.860	−31.294
11	5288725	*N*‐Methyl‐l‐alanine	−5.024	−17.973	−21.447
12	ID C4430471	3,4‐Methylenedioxybenzyl isothiocyanate	−4.769	−28.117	−37.427
13	543323	Rhodinol	−4.528	−23.290	−23.944
14	ID C26205458	Silane [1,4‐dioxane‐2,5‐diylbis (oxy)] Bis [trimethyl‐,	−4.475	−27.658	−32.586
15	634489	2‐Cholestanone	−4.448	−37.118	−33.461
16	566216	2‐Thiopheneacetic acid	−4.091	−33.674	−44.621
17	259846	Lupeol	−3.697	−27.411	−31.124
18	99574711		−3.554	−30.039	−35.553
19	15610	Methyl nanadecanoate	−3.434	−27.775	−28.745
20	5317844	α‐Guaiene	−3.426	−20.825	−17.263
21	6440	2,2‐Dimethyl‐1,3‐butanediol	−3.355	−13.512	−9.837
22	553105	Trimethylsilyl 2‐trimethylsiloxyethyl sulfide	−3.296	−19.519	−21.646
23	94275	δ‐Guaiene	−3.228	−23.892	−22.996
24	5281520	Humulene	−3.017	−21.366	−22.118
25	292285	Octadecane	−2.997	−26.260	−29.446
26	519743	Seychellene	−2.887	−14.604	−16.723
27	57373805	3,5‐Dichloro‐6‐nitrocholestane	−2.825	−21.192	−27.027
28	7461	γ‐Terpinene	−2.619	−19.748	−23.770
29	6918391	β‐Elemene	−2.604	−19.766	−18.670
30	481107734	β‐Sitosterol	−2.432	−77.899	−19.715
31	138332	2‐Bromo‐6‐methylheptane	−2.406	−20.973	−23.606
32	296566	Heptadecane, 9‐hexyl‐	−1.183	−29.177	−31.813
33	93099	3‐Methoxy‐2,2‐dimethyloxirane	−2.343	−11.237	−12.325
34	5281515	Caryophyllene	−2.275	−14.455	−16.819
35	91693075	Carbonic acid, isobutyl 2‐methylbutyl ester	−2.028	−21.294	−22.779

**TABLE 2:** ADME profile of the selected compounds.

S. no.	Compound ID	Mol. W^a^	Qplog o/w^b^	QPP Caco^c^	Qplog hERG^d^	% human oral absorption^e^
1	5280863	286.24	1.03	59.93	−4.99	64.84
2	445639	282.46	5.82	315.88	−3.32	92.84
3	31401	392.57	3.75	41.76	−2.05	77.93

^a^Molecular weight (reasonable value < 500).

^b^Predicted octonal/water partition coefficient (reasonable value from −2.0 to 6.5).

^c^Predicted apparent Caco‐2 cell permeability in nm/s (reasonable value > 25).

^d^Predicted blockage of hERG K^+^ channel (reasonable value < −7).

^e^Percentage of human oral absorption (< 25% is weak and > 80% is strong).

### 2.3 Molecular Dynamics Simulation

**FIGURE 3:**
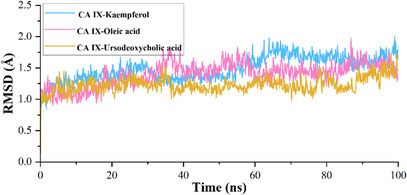
Root mean square deviation of protein–ligand complex during the simulation time of 100 ns.

**FIGURE 4:**
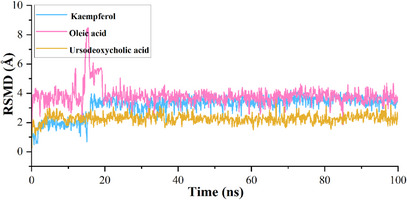
Root mean square deviation of ligand molecules inside the binding site of the protein during the MD simulation.

**FIGURE 5:**
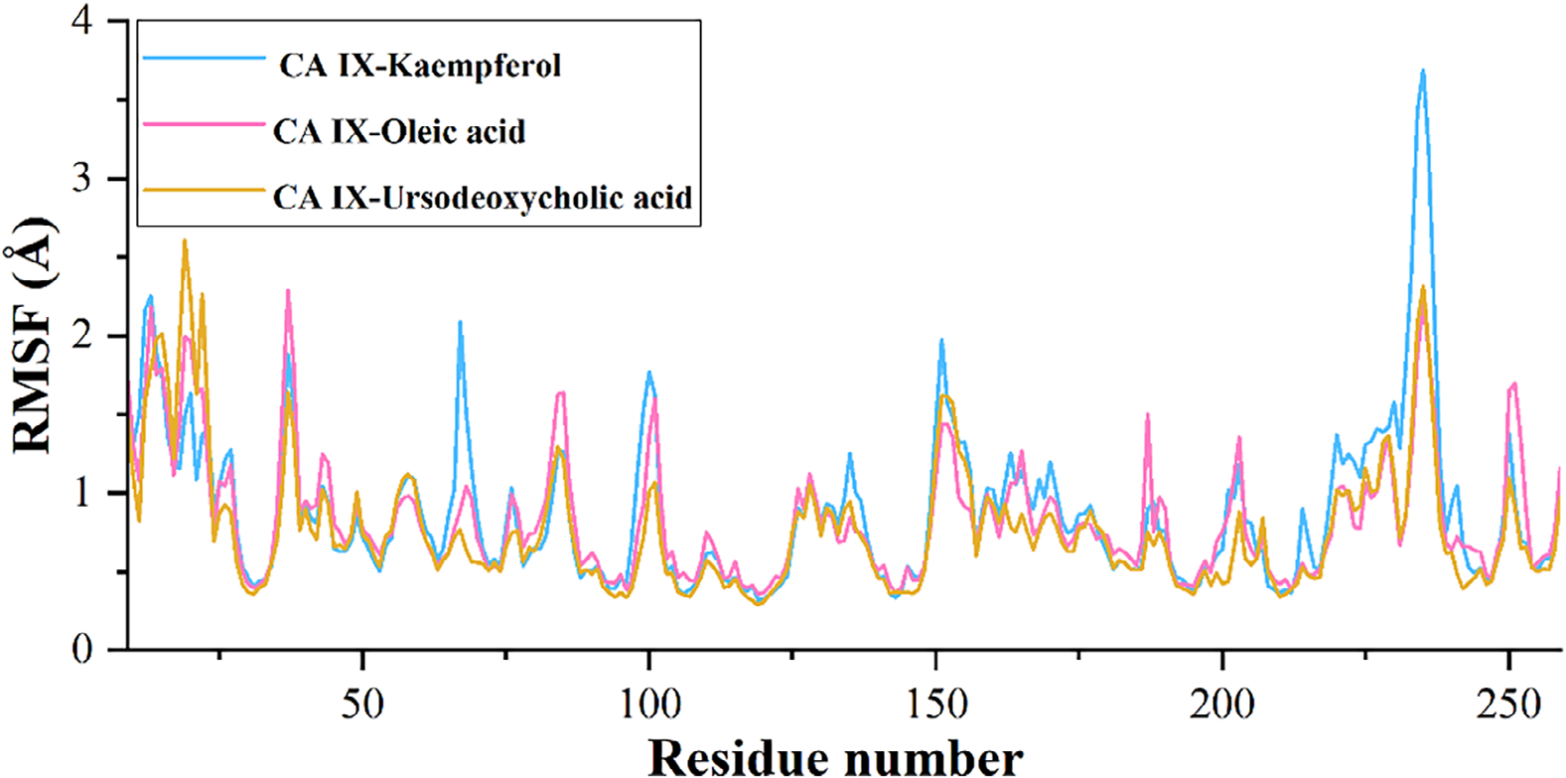
Root mean square fluctuation of the protein–ligand complex, indicating the local fluctuations that occurred during the simulation; the height of the peak represents the fluctuation rate.

**FIGURE 6:**
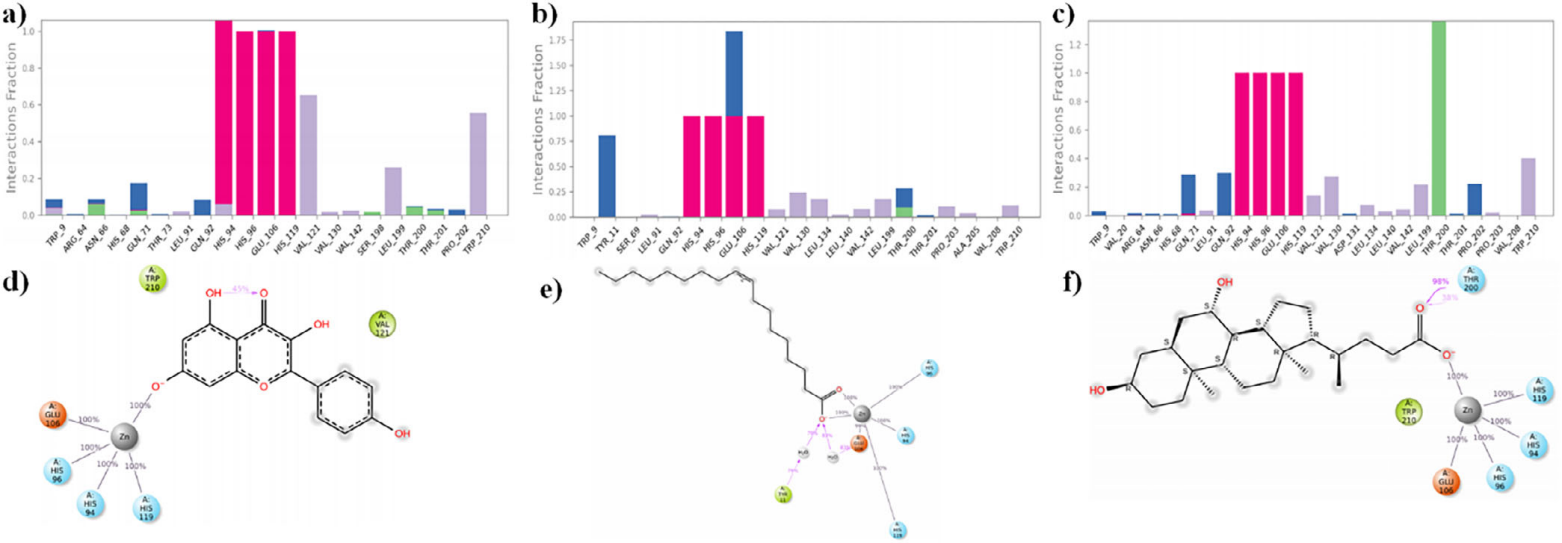
(a–c) The figure represents the type of interactions that were mediated during the simulation time; the green, blue, pink, and purple colors in the bar diagram indicate the H‐bond, water bridge, ionic, and hydrophobic interactions. (d–f) A two‐dimensional figure that represents the percentage of H‐bond interactions that were mediated during the simulation period of 100 ns. Here, the pink and gray colors represent the H‐bond and metal coordination.

The calculated root mean square deviation (RMSD) for CA IX complexed with kaempferol indicates that the initial deviation in the RMSD was observed from 0 to 1.25 Å up to 20 ns, whereas a sudden rise in RMSD was observed after 20 ns and then RMSD falls back to 1.20 Å still 60 ns and a further rise in RMSD was observed to 1.75 Å at 62 ns, which may due to the equilibration phase of the system. Subsequently, a stable RMSD was observed from 62 ns to the end of the 100 ns molecular dynamics (MD) simulation (Figure 3). In the case of ligand RMSD, an increase in RMSD was observed from 0 to 3.5 Å up to 20 ns, after which a stable RMSD was observed throughout the MD simulation, indicating that the ligand molecule bound strongly to the protein‐binding site (Figure 4). The average RMSD and SD values for the protein–ligand complex were found to be 1.75 and 0.20 Å, respectively. The C‐ and N‐terminal regions of the protein showed higher fluctuations compared to other regions in the CA IX–kaempferol complex. The amino acid residues, including Gly12 to Asp14, showed a fluctuation rate of 2.22–2.02 Å, Gly233 to Ser237 a fluctuation rate of about 3.58–2.08 Å (Figure 5). The carbonyl group of the ligand shows metal coordination with Zn at approximately 100% of the simulation frame. In addition, zinc mediates metal coordination with four amino acids, namely Glu106, His96, His94, and His119, around 100% of the simulation period (Figure 6a,d). In the case of the CA IX–oleic acid complex, we noticed many ups and downs in the RMSD values, and the complex was found stabilized with the RMSD value around 1.50 Å, with the least SD value of 0.13 Å (Figure 3). In the case of ligand RMSD, an initial rise was observed up to 18 ns with an RMSD value of approximately 3.5 Å, after which a sudden spike in the RMSD for a short time was observed from the 18–20 ns simulation, after which the complex was found stabilized around 3.5 Å at the end of the simulation period (Figure 4). Three amino acid residues, including Arg19 (2.05 Å), Gly37 (2.22 Å), and Gly235 (2.24 Å), showed higher fluctuation in the CA IX–oleic acid complex (Figure 5). The carbonyl group present in oleic acid showed stable and strong metal coordination with His96, His94, His119, and Glu106 at approximately 100% of the simulation frame. The carbonyl group of oleic acid also showed two H‐bond interactions with water molecules (79% and 83%), and two water‐mediated H‐bonds with Tyr11 (79%) and Glu106 (83%) (Figure 6b,e). Many ups and downs in the RMSD were observed in CA IX‐ursodeoxycholic acid and the average RMSD and SD values were found to be 1.2 and 0.02 Å, respectively (Figure 3). A stable RMSD of 2.0 Å was observed for the ligand (Figure 4). Four amino acid residues, such as Pro18 (2.07 Å), Arg19 (2.60 Å), Val20 (2.23 Å), and Gly235 (2.20 Å), showed higher fluctuations in the complex (Figure 5). A similar type of ionic interaction and metal coordination as observed in the CA IX–oleic acid was noticed here. In addition, the carbonyl group of ursodeoxycholic acid showed two H‐bond interactions with Thr200 (98% and 38%, respectively) (Figure 6c,f). The Desmond MD results demonstrate that the observed residues play a vital role in the binding of ligand molecules to the binding site of human CA IX.

### 2.4 Density Functional Theory Analysis

**FIGURE 7:**
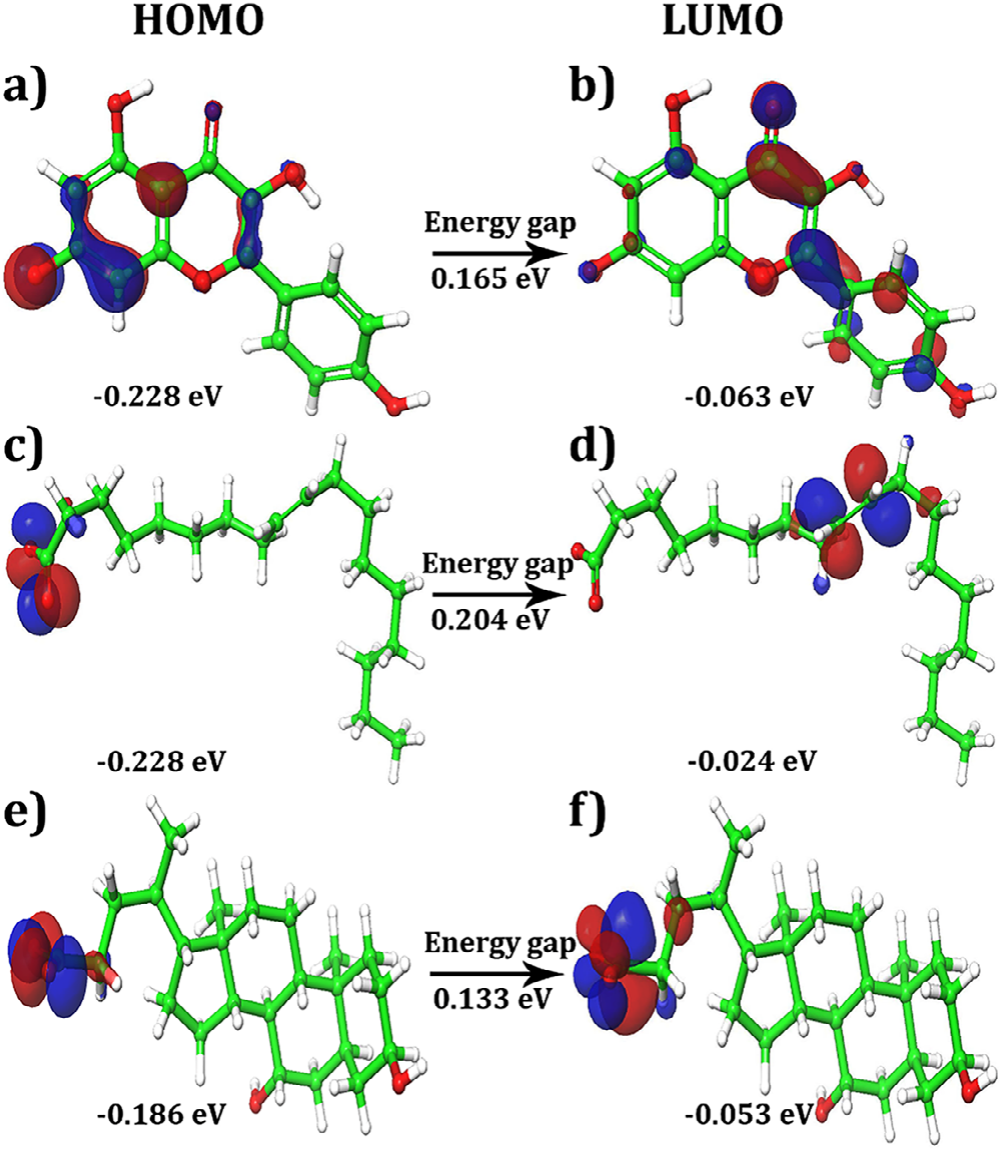
(a–f) HOMO, LUMO distributions in the selected molecules. (a, b) Kaempferol, (c, d) oleic acid, (e, f) ursodeoxycholic acid.

The highest occupied molecular orbital (HOMO)–lowest unoccupied molecular orbital (LUMO) map and gap values between the HOMO and LUMO are presented in Figure 7a–f and Table 3, respectively. These values provide insights into the electronic properties of lead molecules. The HOMO values ranged from −0.186 to −0.228 eV, while the LUMO values ranged from −0.024 to −0.063 eV. The HOMO and LUMO energies suggested that the ligand molecules had a strong affinity for the protein. In addition, the energy gap between the HOMO and LUMO, known as the HLG (HOMO–LUMO gap), was calculated. HLG is a measure of the chemical stability and reactivity of the lead molecules. A small HLG value indicates that lead molecules are highly reactive, stable, and polarized. The calculated values of HLG for the selected lead molecules ranged from 0.133 to 0.204 eV, suggesting good reactivity and stability.

**TABLE 3:** HOMO, LUMO, and energy gap values of the compounds.

S. no	Compound ID	HOMO (eV)	LUMO (eV)	Energy gap (eV)
1	5280863	−0.18	−0.05	0.13
2	445639	−0.22	−0.02	0.24
3	31401	−0.22	−0.06	0.16

**FIGURE 8:**
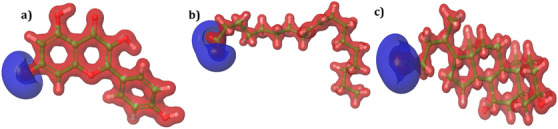
(a–c) Molecular electronic surface potential. Positive charges in the compounds are represented in blue color, while negative charges are represented in red color. (a) Kaempferol, (b) oleic acid, (c) ursodeocycholic acid.

The HOMO and LUMO energies of the lead molecules are distributed in two distinct regions. For kaempferol, HOMO densities were observed at the O^−^ and C─H positions of the 3,5‐dihydroxyl‐4‐oxo‐4*H*‐1 benzopyron‐7‐olate group (Figure 7a). On the other hand, LUMO densities were observed in almost the entire structure of the molecule, indicating high reactivity (Figure 7b). In compound oleic acid, the HOMO energies were observed at the O^−^ and H positions of the acetyl group (Figure 7c), whereas the LUMO densities were observed at the H position of the (3*Z*)‐hex‐3‐ene group (Figure 7d). For ursodeoxycholic acid, the HOMO map showed densities at the O^−^ and H positions of the acetate moiety (Figure 7e). Similarly, LUMO densities were observed at the same position, with an additional H position (Figure 7f). In all compounds, the ─OH position showed a positive charge with a blue color, while the rest of the positions possessed a negative charge and are shown in red (Figure 8a–c).

### 2.5 Prime Molecular Mechanics Generalized Born Surface Area Analysis

The calculated molecular mechanics generalized Born surface area (MM/GBSA) scores for the ligand molecules are listed in Table 4. The ursodeoxycholic acid exhibited a score of −20.24 kcal/mol, oleic acid displayed a score of −20.30 kcal/mol, and the highest binding free energy was observed for kaempferol, which exhibited a score of −25.58 kcal/mol. These findings indicate that all lead molecules exhibit a strong binding affinity for the protein receptor, as evidenced by their better binding free energy scores. This suggests that these molecules can interact strongly with the active site of the protein and potentially disrupt its biological activity. This MM/GBSA analysis provided valuable information and indicated its potential as a candidate for further investigation in drug development and other applications.

The main findings of our study are (i) the extract of *Ipomoea obscura* tested in in vitro conditions has revealed its potential inhibitory effect on the inhibition of bladder cancer cell line T24. (ii) The molecular docking and dynamics studies revealed the higher binding affinity of the molecules against the target Carbonic Anhydrase IX (CA IX) [30]. The extract exhibited significant cytotoxic effects specific to cancer cells in the tested cell lines in a time‐ and dose‐dependent manners. Previous studies have shown that *Ipomoea obscura* extracts have anti‐cancer and antioxidant properties [31, 32]. Our study explored the binding affinities of the selective molecules from *I. Obscura* against the CA IX, one of the vital targets in bladder cancer, which aids the cancer cells to sustain hypoxia and acidosis in the microenvironment [33]. In our molecular modeling and simulation studies, all three shortlisted compounds (kaempferol, oleic acid, and ursodeoxycholic acid) were found to maintain stable interaction with the receptor target CA IX. The pharmacological effects of these three molecules have been previously investigated under various conditions and revealed that all three have anti‐cancer properties [34, 35, 36]. A study by Alruhaimi et al. evaluated the binding affinity of coumarins (*Calendula officinalis*) with CA IX through docking and MD simulations and reported that the compound showed favourable binding affinity through hydrophobic interactions and also displayed potential binding free energy [37]. In another study, triazole benzene sulfonamide derivatives have shown potential binding affinity with CA‐IX through in silico analysis [38]. Notably, three amino acid residues, His96, Glu106, and His119, exhibited consistently strong ionic interactions with zinc throughout the MD simulation. Overall, the insights gained from the molecular geometry, electron donor/acceptor characteristics, and HOMO‐LUMO property analysis provided valuable information regarding the interaction and reactivity of the ligand molecules with the protein. The results of MM‐GBSA analysis suggest that these compounds can strongly interact with the active site of the protein, potentially interfering with its biological function.

**TABLE 4:** Molecular docking scoring functions, interaction profile of the selected compounds against human carbonic anhydrase IX.

S. no.	Compound ID	Glide score (kcal/mol)	Glide energy (kcal/mol)	Glide *E*_model_ (kcal/mol)	H‐bond interaction	π–π stacking	π cation	Metal coordination	Salt bridge	Δ*G*_bind_
1.	5280863	−8.86	−33.66	−49.02	—	His94	—	Zn264		−25.58
2.	445639	−6.99	−31.04	−35.27	Thr200	—	—	Zn264	Zn264	−20.30
3.	31401	−6.968	−34.747	−38.202	Thr200, Trp9	—	—	Zn264	Zn264	−25.58

## 3 Conclusion

We evaluated the in vitro cytotoxicity of the ethanolic extract of *I. obscura* against bladder cancer T24 cell lines to determine its anticancer potential. The in vitro analysis showed that the extract inhibited T24 cell lines with an IC_50_ value of 82.10 µg/mL, comparable to that of standard doxorubicin hydrochloride (75.04 µg/mL). The potent anticancer activity may be due to the secondary metabolites present in kaempferol, oleic acid, and ursodeoxycholic acid. The three metabolites showed higher binding affinities toward the human CA IX enzyme. The binding stability of these compounds within the binding site of the CA IX enzyme was re‐scored using Prime MM/GBSA analysis. Furthermore, MD simulations confirmed their stability. In summary, *I. obscura* extract acts as a natural therapeutic agent for cancer patients, and the bioactive constituents from the plants may serve as a promising substitute for oxidative stress and cancers.

## 4 Experimental

### 4.1 Plant Collection

*I. obscura* (L.) Ker Gawl was collected from the Madurai District, Tamil Nadu, India. The plant was authenticated by Dr. G. V. S. Murthy from the Botanical Survey of India, Tamil Nadu Agricultural University Campus, Coimbatore, India. A voucher specimen was deposited in the laboratory for future reference under the code BSI/SRC/5/23/2010‐11/Tech.

### 4.2 Ethanolic Extract Preparation

A total of 300 g of dried leaf powder was extracted with 1500 mL of ethanol in a sporadic shaker for 72 h at room temperature. The extract was collected and concentrated at 40°C under reduced pressure, using a rotary evaporator. The dried extract was then stored in airtight bottles at 4°C for future study. Phytochemical studies in previous reports on *I. obscura* ethanolic leaf extracts have identified a range of secondary metabolites, including flavonoids (e.g., kaempferol), fatty acids (e.g., oleic acid), phenolic acids, and alkaloids such as ipobscurine derivatives. These compounds were not re‐isolated in the present study; instead, we focused on screening reported constituents through computational docking and dynamics studies to elucidate their interaction with the CA IX enzyme.

### 4.3 Cytotoxicity Analysis

Human T24 bladder cancer cell lines were obtained from the National Center for Cell Science (NCCS) in Pune. The cells were cultured in a 75 cm^2^ flask containing Dulbecco's modified Eagle's medium (DMEM) supplemented with 10% fetal bovine serum and antibiotics and allowed to reach 80% confluence. Once the cells reached confluence, the medium was removed and the cells were washed once with phosphate‐buffered saline (PBS). A Trypsin (0.25%)‐EDTA solution was then added and incubated for 3–5 min at 37°C. Fresh medium (with serum) was introduced and the cells were gently dispersed using a pipette. A known number of cells was then transferred to new flasks or microtiter plates for further experiments. The cells were incubated at 37°C in a 5% CO_2_ atmosphere. Although cytotoxicity was assessed at all four time points, the IC_50_ values were specifically calculated for the 48‐h incubation period, which showed the most consistent and interpretable dose‐response trends.

#### 4.3.1 MTT‐Cell Proliferation Assay

Cell growth inhibition was determined using an MTT assay [39]. The 3‐(4, 5‐dimethylthiazol‐2‐yl)‐2, 5‐diphenyltetrazolium bromide (MTT) assay is a straightforward, nonradioactive colorimetric method used to evaluate cell cytotoxicity, proliferation, and viability. Cells were seeded in 96‐well plates at a density of 5000 cells/well and cultured for 1 day. They were then treated with various concentrations of *I. obscura* for 12, 24, 48, and 72 h at 37°C in a 5% CO_2_ atmosphere. Control wells were incubated with medium only in the same 96‐well plates. At the end of the incubation period, the medium was removed, and MTT (5 mg/mL) was added, followed by an additional 4‐h incubation. The medium was then discarded, and DMSO was added to each well to dissolve the formazan crystals. Absorbance was measured at 570 nm using a microtiter enzyme‐linked immunosorbent assay (ELISA) plate reader. All experiments for the extract were conducted in triplicate, including untreated cell controls and blank cell‐free controls. Cell viability was expressed as a percentage of the control.

### 4.4 Ligand Preparation

Phytochemicals related to *I. obscura* were obtained from previously published studies [19, 21, 40, 41, 42]. The 2D (Two‐Dimensional Structure Data File) chemical structures of the compounds were sourced from the PubChem Database (https://pubchem.ncbi.nlm.nih.gov/). The compounds were subsequently prepared using the LigPrep module of Schrödinger, with default settings [43]. LigPrep first transforms the 2D structures into three‐dimensional (3D) structures, ensuring correct chiral configurations by adding hydrogen atoms and adjusting the bond orders. The ionization and tautomerization states of the ligand were established at a physiological pH of 7.2 ± 2.0, using the Epik module in Schrödinger. Subsequently, the ligands were optimized and minimized using an OPLS3 force field [44].

### 4.5 Protein Preparation

The 3D structure of human CA IX in complex with 5‐(1‐naphthalen‐1‐yl‐1,2,3‐triazol‐4‐yl) thiphene‐2‐sulfonamide was obtained from the Protein Data Bank (PDB ID: 5FL4) [45]. The obtained protein was prepared using the Protein Preparation Wizard of Schrödinger [46]. The protein preparation has three components: pre‐processing, review, modification, and refinement. In the pre‐processing step, the correct bond orders for the protein were assigned, the addition of hydrogen atoms, zero‐bond orders to metals were created, disulfide bonds were created, missing side chains were modeled, water molecules beyond 5 Å from the het groups were deleted, and finally the het states were generated using the Epik module by utilizing the physiological pH of 7.0 ± 2.0. In the review and modification step, unwanted water molecules that do not interact properly with the protein were removed. In the third step (refinement), optimization and restrained minimization were performed until the RMSD of the non‐hydrogen atoms reached 3.0 Å.

### 4.6 Molecular Docking Analysis

Molecular docking studies were performed to determine the binding interactions of small molecules with the active site of human CA IX. Chain A (PDB ID: 5FL4) co‐crystallized with 5‐(1‐naphthalen‐1‐yl‐1,2,3‐triazol‐4‐yl‐thiphene‐2‐sulfonamide) was selected for docking studies, and the remaining chains B, C, and D were initially deleted during the protein preparation process. The grid was generated using the Receptor Grid Generation panel by selecting the co‐crystallized ligand. Finally, the Glide (XP) extra precision mode was employed to perform the docking process using the default setting [47, 48]. The ranking of the ligand molecules was based on Glide scoring functions (Chem score).

### 4.7 MD Simulation

The protein–ligand complex dynamic simulation was performed using Desmond v3.0, implemented in the Schrödinger package [49]. Initial minimization was performed to eliminate high‐energy intramolecular interactions in the system. The protein–ligand complex was solvated in a cubic simulation box in a single‐point charge (SPC) water environment [50]. The system was neutralized by adding appropriate counter ions (Na^+^ or Cl^−^) and replacing the water molecules to balance the net charge of the system. The system was minimized using the OPLS_2005 force field [51]. To control the pressure and temperature, the Martyna–Tuckerman–Klein chain coupling and Nose–Hoover chain coupling schemes were used [52]. r‐RESPA integrator and particle mesh Ewald methods were used to calculate the non‐bonded forces and long‐range electrostatic interactions [53]. The cut‐off radius in the Coulomb interactions was 9.0 Å. Finally, in the NPT ensemble, the simulation was carried out at a temperature of 300 K and a pressure of 1 bar. All simulations were carried out for 100 ns, and the trajectories were saved at 4.8 ps intervals. The simulation interaction diagram module implemented in Schrödinger was used to ascertain the interaction profile between the protein and the ligand. Parameters, including RMSD, root mean square fluctuation (RMSF), and interaction profiles, were monitored, which in turn determined the stability of the ligand in the active site of human CA IX.

### 4.8 Density Functional Theory Calculation

Density functional theory (DFT) is a computational approach used to describe the electronic properties of molecules at the quantum‐mechanical level. It provides valuable insights into the electrostatic map, electron density, and frontier molecular orbitals (such as the HOMO and LUMO) of ligand molecules. These electronic features help in understanding biological activity, molecular characteristics, binding affinity, and chemical reactivity with proteins. In this study, DFT was employed using a hybrid DFT approach, specifically, the B3LYP functional. The B3LYP functional combines Becke's three‐parameter exchange potential with the Lee–Yang–Parr correlation function [54]. The calculations were performed using the 6‐31G* basis set, which includes the necessary basis functions to accurately describe the molecular electronic structure. All DFT calculations were performed using Jaguar [55].

### 4.9 Prime MM/GBSA Calculation

To determine the binding affinity between the protein and ligand complexes, binding free‐energy calculations were performed using Prime MM/GBSA. The binding free energy was calculated using the following equation:

$$\Delta G_{bind}=\Delta E_{MM}+\Delta G_{SGB}+\Delta G_{SA}$$

Δ*E*_MM_ minimizes gas‐phase molecular mechanics energies, which involves optimizing the molecular conformations of the protein–ligand complex, protein, and unbound ligand to account for their interactions and achieve the most stable configurations. The SGB is a solvation model for polar solvation (*G*_SGB_) with default parameters. Δ*G*_SA_ is a solvent‐accessible surface area (SASA) composed of non‐polar surface area and van der Waals interactions. *G*_complex_, *G*_protein_, and *G*_ligand_ minimize the energy of the protein–ligand complex, protein energy, and unbound ligand energy, respectively [56].

### 4.10 Statistical Analysis

The results (mean ± SD) of cell proliferation and invasion were subjected to statistical analysis by Student's *t*‐test for comparison with the standard drug. The level of significance was set at *p* < 0.05. All experiments were repeated twice using triplicate samples.

## Author Contributions

**Saravana Prabha Poochi**: conceptualization, methodology, investigation, data curation, writing of original draft. **Rajamanikandan Sundarraj**: software, validation, review, and editing. **Manikandan Vani Raju**: resources: drafting of the article. **Meenakshi Kaniyur Chandrasekaran**: resources: drafting the article. **Rathi Muthaiyan Ahalliya**: resources, review, and editing. **Chella Perumal Palanisamy**: software, validation, review, and editing. **Gopalakrishnan Velliyur Kanniappan**: conceptualization, methodology, supervision, project administration, validation, writing – review and editing. **Benod Kumar Kondapavuluri** and **Muthu Thiruvengadam**: resources, software, validation, review, and editing.

## Conflicts of Interest

The authors declare no conflicts of interest.

## Data Availability

The data that support the findings of this study are available from the corresponding author upon reasonable request.
